# A Low Glycaemic Index Diet in Pregnancy Induces DNA Methylation Variation in Blood of Newborns: Results from the ROLO Randomised Controlled Trial

**DOI:** 10.3390/nu10040455

**Published:** 2018-04-06

**Authors:** Aisling A. Geraghty, Alexandra Sexton-Oates, Eileen C. O’Brien, Goiuri Alberdi, Peter Fransquet, Richard Saffery, Fionnuala M. McAuliffe

**Affiliations:** 1 UCD Perinatal Research Centre, Obstetrics and Gynaecology, School of Medicine, University College Dublin, National Maternity Hospital, Dublin 2, Ireland; aisling.geraghty@ucdconnect.ie (A.A.G.); eileen.obrien@ucd.ie (E.C.O.); goiuri.alberdi@ucd.ie (G.A.); 2 Cancer and Disease Epigenetics, Murdoch Children’s Research Institute, Melbourne, Victoria 3052, Australia; alex.sextonoates@mcri.edu.au (A.S.-O.); peter.fransquet@mcri.edu.au (P.F.); richard.saffery@mcri.edu.au (R.S.); 3 Department of Paediatrics, University of Melbourne, Victoria 3010, Australia

**Keywords:** epigenetics, DNA methylation, programming, methylome, fetus, pregnancy, intervention, diet, glycaemic index

## Abstract

The epigenetic profile of the developing fetus is sensitive to environmental influence. Maternal diet has been shown to influence DNA methylation patterns in offspring, but research in humans is limited. We investigated the impact of a low glycaemic index dietary intervention during pregnancy on offspring DNA methylation patterns using a genome-wide methylation approach. Sixty neonates were selected from the ROLO (Randomised cOntrol trial of LOw glycaemic index diet to prevent macrosomia) study: 30 neonates from the low glycaemic index intervention arm and 30 from the control, whose mothers received no specific dietary advice. DNA methylation was investigated in 771,484 CpG sites in free DNA from cord blood serum. Principal component analysis and linear regression were carried out comparing the intervention and control groups. Gene clustering and pathway analysis were also explored. Widespread variation was identified in the newborns exposed to the dietary intervention, accounting for 11% of the total level of DNA methylation variation within the dataset. No association was found with maternal early-pregnancy body mass index (BMI), infant sex, or birthweight. Pathway analysis identified common influences of the intervention on gene clusters plausibly linked to pathways targeted by the intervention, including cardiac and immune functioning. Analysis in 60 additional samples from the ROLO study failed to replicate the original findings. Using a modest-sized discovery sample, we identified preliminary evidence of differential methylation in progeny of mothers exposed to a dietary intervention during pregnancy.

## 1. Introduction

The in utero environment is key to healthy fetal development and recent research has highlighted how the developing fetus is sensitive to environmental influence, potentially mediated by epigenetic variation [1]. Mounting evidence supports a role for variable DNA methylation, established very early in life, in the process of ‘programming’ of risk for a variety of common non-communicable diseases such as cardiovascular disease and type-2 diabetes mellitus [2,3].

Adverse maternal environments during pregnancy, such as those seen with maternal obesity or gestational diabetes mellitus, have been associated not only with increased risk of infant mortality, but also with increased lifelong risks of offspring obesity and metabolic and cardiovascular dysfunction in adulthood [4,5]. Exposure to gestational diabetes has been shown to induce changes in DNA methylation patterns in offspring placental tissue and cord blood [6], highlighting a potential mechanism by which environment in pregnancy can influence gene functioning and development of metabolic diseases of the offspring in later life. Specifically looking at hyperglycaemia-induced epigenetic changes, results from an adult mouse model (with normal glucose control) identified dramatic and long-lasting effects that transient hyperglycaemic spikes can have on vascular cells and epigenetic profiles, even in a non-diabetic cohort [7]. Exposure to these epigenetic marks in utero may influence the development of metabolic diseases of the offspring. 

Extensive associations have been shown between dietary factors and alterations in the epigenome in adults, which in turn impacts the individual’s health [8], however, there is a paucity of research on this in relation to pregnancy. Research to date has focused on epidemiological studies and has identified a tentative link between the maternal diet during pregnancy and early postnatal nutrition in altering methylation patterns in the offspring [9,10]. Such dietary exposures, or nutrient insufficiencies, can influence the offspring through developmental programming of diseases both in childhood and in later life [10,11]. The genomic regions in human offspring that may be sensitive to maternal dietary exposures during in utero development have not been well characterised. Maternal diet influencing individual DNA methylation patterns in animal offspring have been clearly shown, but research in humans is generally limited to observational cohort studies with poorly defined exposures [12]. Intervention studies, particularly on diet during pregnancy as assessed by Epigenome Wide Association Studies (EWAS), are scarce. One such study involving a randomised controlled trial of maternal n-3 polyunsaturated fatty acids supplementation found no strong effects on CD4+ T-cell methylation profiles in the offspring [13]. Further intervention studies like these are required to identify sensitive, modifiable regions in the infant epigenome.

As epigenetics marks influence future health outcomes and are potentially modifiable, thereby providing novel targets for intervention, we sought to investigate the effects of an intervention targeting maternal diet and glycaemic control in pregnancy on offspring epigenetic profile. To determine whether exposure to a low glycaemic index diet during pregnancy could alter the epigenome of offspring, we selected mother and neonate dyads from a previously conducted randomised controlled trial and compared genome-wide DNA methylation profiles using cell-free DNA from neonatal cord blood serum. We hypothesised that a prenatal maternal low glycaemic index diet would modulate DNA methylation patterns in the neonate.

## 2. Materials and Methods

### 2.1. Study Population

The study sample comprised 60 sex-matched neonates from a large prospective mother-child birth cohort of the ROLO study (Randomised cOntrol trial of LOw glycaemic index diet versus no dietary intervention to prevent recurrence of fetal macrosomia), with 30 participants from the intervention arm and 30 participants from the control. The study was carried out in Dublin, Ireland, trial registration: ISRCTN54392969. Secundigravid pregnant women over 18 years of age, who had previously given birth to a macrosomic infant (birth weight > 4 kg), were recruited before the 18th week of gestation. Exclusion criteria included any underlying medical disorders requiring medication, multiple pregnancy, or those with a previous history of gestational diabetes. All subjects gave their informed written consent for inclusion before they participated in the study. The study was conducted in accordance with the Declaration of Helsinki, and institutional ethical approval was granted by the National Maternity Hospital. After obtaining written consent, participants were randomised into either the control or intervention arm of the study. The control group received no specific dietary advice, just routine antenatal care, while the intervention arm received dietary advice regarding healthy eating and, specifically, about a low glycaemic index diet which they were advised to follow for the duration of the pregnancy. Further details and methodology for the study have been previously published [14]. Height and weight were taken at the first visit (approx. 14 weeks gestation) by a trained healthcare professional and early-pregnancy body mass index (BMI, kg/m^2^) was calculated. Detailed health and lifestyle questionnaires were also collected [14]. Three-day food diaries were completed in each trimester of pregnancy, one before the intervention and two after. These diaries were completed on three consecutive days (including two weekdays and a weekend day) corresponding to trimester one, two, and three and were returned to the research team at subsequent hospital visits. The food diaries were entered by the research dietitian into nutritional analysis software NetWISP version 3.0 (Tinuviel software, Llanfechell, Anglesey, UK). This software utilises the food composition database from the 6th edition of McCance and Widdowson’s food composition tables [15]. Dietary glycaemic index in each trimester was calculated using NetWISP version 3.0 based on values using the 2008 International Tables of Glycaemic Index Values and more recently published values [16]. A sample of cord blood was collected from the umbilical cord within 5 min of delivery of the infant and serum was stored at −80 °C. The 60 participants were selected due to availability of a sample and among these they were matched for child sex and intervention and control group. Those with the highest DNA yields were selected for the discovery analysis.

### 2.2. Dietary Intervention

The intervention consisted of one group dietary education session with a research dietitian, lasting approximately two hours. The education session consisted firstly of general healthy eating guidelines for pregnancy and how to follow the food pyramid, then the participants were educated specifically about the glycaemic index. The advice involved choosing as many low glycaemic index foods as possible and replacing high glycaemic index foods with low glycaemic index alternatives. The recommended diet was eucaloric and participants were not advised to reduce their current calorie intakes. After the education session, the participants received written resources about healthy eating and low glycaemic index foods and they met with the dietitian again at 28 and 34 weeks gestation to reinforce the diet and to answer any questions they had. Results of the intervention on maternal dietary intakes have been previously published [17].

### 2.3. DNA Extraction and Genome-Wide Methylation Detection

Circulating cell-free DNA (cfDNA) was extracted from cord blood serum samples using the ChargeSwitch^®^ gDNA 1 mL Serum Kit (©2005 Invitrogen Corporation, California, CA, USA). The purified genomic DNA was then bisulfite converted (EZ-96 DNA Methylation-Lightning™ MagPrep kit, Irvine, CA, USA), and DNA methylation was measured in over 850,000 CpG sites using the Illumina Infinum MethylationEPIC BeadChip Array (HM850, Illumina, San Diego, CA, USA), carried out by ServiceXS in Leiden, The Netherlands. Data was processed using the minfi package for R and normalized using Subset-quantile within array normalization (SWAN) [18]. Probes on the X and Y chromosomes, those associated with single-nucleotide polymorphisms (SNPs) (minor allele frequency > 1%), and those cross-reactive [19], or which failed in one or more samples were removed, leaving data for 771,484 probes common to all samples for subsequent analysis.

### 2.4. Statistical Analysis

Principal component and linear regression analysis was performed using the weighted gene co-expression network analysis (WGCNA) and limma packages for R [20,21]. Regression analyses were used to identify regions of methylation associated with maternal and fetal factors and exposure to the dietary intervention using limma. This analysis was adjusted for confounders (8 HM850 array chips, HM850 array chip position, sex, and gestational age) identified through principal components analysis using WGCNA for R statistical package. The Benjamini-Hochberg False-Discovery-Rate method [22] was used to adjust for multiple testing when defining statistically significant differentially methylated probes. Gene ontology analysis was carried out on annotated genes associated with the top 1000 differentially methylated CpG sites between intervention and control groups using DAVID (The Database for Annotation, Visualization and Integrated Discovery v6.87, Leidos Biomedical Research Inc., Frederick, MA, USA) [23,24] and functional clusters were created using Kyoto Encyclopedia of Genes and Genomes (KEGG) and Reactome Pathways. M-values were used to perform statistical analysis (principal component analysis and linear regression modelling), as beta values are known to be severely heteroscedastic at highly hyper and hypomethylated regions [25]. Beta values and SEQUENOM methylation levels were used to visualise sample DNA methylation levels and distributions. We predicted cellular composition for cord blood based on DNA methylation profiles in the cell-free DNA from the serum samples. The relative proportion of cell types within each sample was estimated using the minfi function estimateCellCounts and cord blood as the composite cell type. Differences in cell composition between the control and intervention group were investigated using Mann-Whitney U tests using SPSS (Statistical Package for the Social Sciences) software version 24.0 (IBM, Armonk, NY, USA).

### 2.5. Replication

Following analysis of the genome-wide methylation data, three candidate genes were selected for validation and internal replication by the Sequenom MassARRAY EpiTYPER platform (Sequenom, Agena Biosciences, San Diego, CA, USA). These included Interleukin 17D (*IL17D*), Nuclear Factor I C (*NFIC*), and Tubulin Folding Cofactor D (*TBCD*). Criteria for selection included having multiple CpG sites within the top 1000 highest ranked probes associated with intervention/control group, and previous evidence for a role in growth and/or metabolism. Each locus-specific assay covered one HM850 CpG probe site (Table S1—Supplementary Material) and assays were designed using DNA sequences extracted from the University of California Santa Cruz UCSC Genome Browser (hg 19) and EpiDesigner (epidesigner.com).

Replication was carried out in a further subset of 60 sex- and group-matched neonatal cord blood serum samples from the ROLO study, making a total sample size of 120 for whom methylation at specific genes was available. These 60 participants were selected again due to availability of a sample and among these they were matched for child sex and intervention and control group. Those with the highest DNA yields were selected for the replication analysis. DNA underwent bisulphite conversion with EZ-96 DNA Methylation-Lightning MagPrep kit (Irvine, CA, USA), followed by polymerase chain reaction (PCR) amplification in triplicate. Using Sequenom MassARRAY, methylation values were obtained for CpG sites from Sequenom EpiTYPER data output. Triplicate samples were used to create an average methylation level at each CpG data point, excluding any triplicate value outside 10% of the median for that point. This was carried out using STATA14 statistical software (StataCorp LP. Stata Statistical Software: Release 14. College Station, TX, US). Independent samples *t*-tests were used to compare differences based on intervention or control group. 

## 3. Results

### 3.1. Cohort Characteristics and the Dietary Intervention

The characteristics of the control and intervention groups are comparable, with no significant differences in maternal age, weight, or education level, neonate birthweight or gestational age (see Table 1). There was no difference in mean daily glycaemic index prior to the intervention being implemented in the first trimester between the groups (57.7 intervention vs. 57.5 control, *p* = 0.79). However, there was a significant difference in glycaemic index in the third trimester (Mean: 55.3, Standard Deviation (SD): 3.8 in the intervention group vs. mean: 57.4, SD: 3.0 in the control, *p* = 0.03), which was also associated with a significant reduction in dietary glycaemic index from trimester one to trimester three (*p* = 0.001) in the intervention group only. Distributions of BMI categories for the mothers also differed with more normal-weight women being in the control group (*p* = 0.04).

### 3.2. Principal Component Analysis

**Figure 1:**
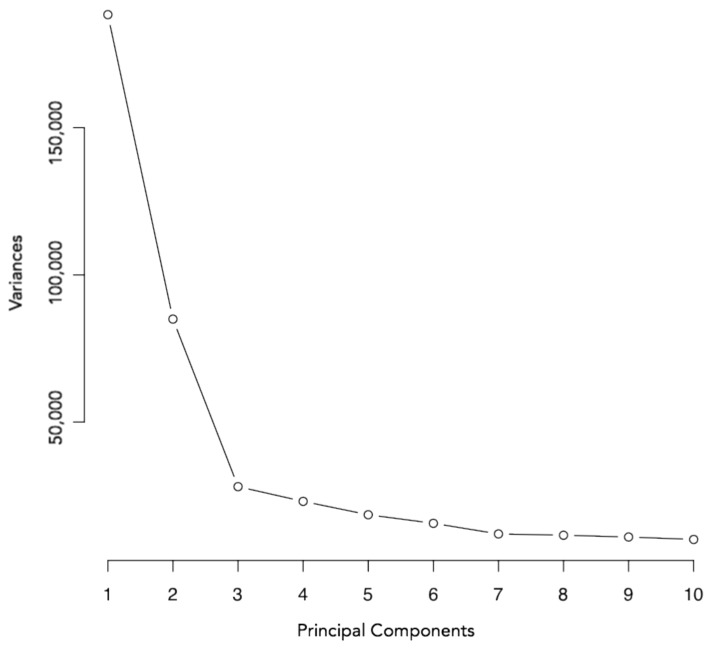
Scree plot generated with M-values for 771,484 probes on the HM850 array. Variance is shown on the y-axis; principal components are shown on the x-axis.

Principal component analysis was conducted initially in order to examine sources of variation within the HM850 methylation dataset. This revealed that position on the array chip, intervention status, array chip number, neonate sex, and gestational age were contributing to the majority of variation within the data (Figure 1). Importantly, intervention status was associated with the second largest proportion of variance in cord blood DNA methylation levels, which accounted for 11.02% of total variation within the dataset. There were no differences in distribution of the control or intervention samples on the chip or chip position (*p* = 0.109, *p* = 0.975, respectively, Fisher’s Exact Test). Neither maternal weight or BMI, nor neonatal birth weight were associated with DNA methylation patterns (Table 2). 

### 3.3. Linear Regression Analysis

Linear regression analysis was used to identify specific differentially methylated probes according to intervention group. This took into account variation associated with the following covariates: HM850 array chip, HM850 array chip position, sex, and gestational age. This analysis identified a total of 28,997 probes (unadjusted *p* ≤ 0.01) between the intervention and control groups. However, despite clear evidence that DNA methylation profile was strongly predicted by intervention group (Figure 1), no individual probes remained significantly differentially methylated between the intervention and control groups after adjusting for multiple testing (adjusted *p* ≤ 0.05).

**Figure 2:**
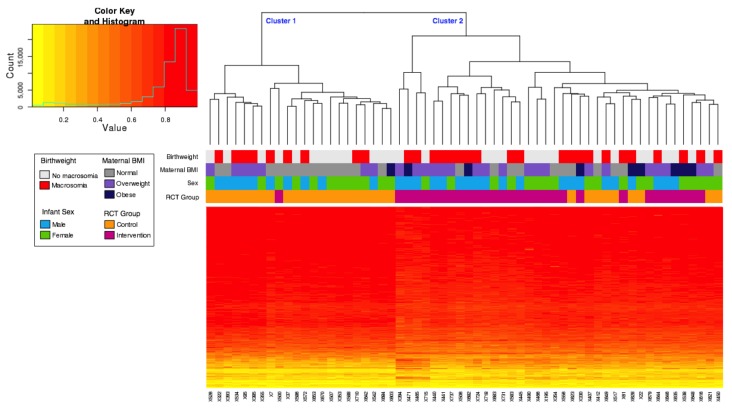
Hierarchical clustering and heatmap of HM850 methylation values of the top 1000 probes associated with intervention/control. Adjusted for infant sex, gestational age, chip, and chip position. The histogram depicts the distribution of methylation levels across all samples and probes, the beta value is plotted on the x-axis and number of probes on the y-axis. RCT: Randomised Controlled Trial.

Nevertheless, hierarchical clustering of the top 1000 unadjusted differentially methylated probes revealed two major methylation clusters associated with the intervention study (Figure 2), with 70% (21/30) of neonates born to mothers in the control group in cluster 1, and 97% (29/30) of neonates born to mothers in the intervention group in cluster 2 (*p* ≤ 0.01, Fisher’s Exact Test). Neither sex nor the presence of macrosomia tracked with these clusters (Figure 2), however, maternal BMI categories were significantly different between the two clusters (*p* = 0.03, Fisher’s Exact Test), with significantly more mothers in the normal category in cluster 1. Participants were split into tertiles based on trimester 3 dietary Glycaemic Index (GI) which resulted in the following distributions: cluster 1 contained 6 Low GI, 8 Medium GI, and 8 High GI and cluster 2 contained 14 Low GI, 12 Medium GI, and 12 High GI. There was no association of GI tertile with the two methylation clusters (*p* = 0.840, Fisher’s Exact Test).

For all probes, the average beta value in the control samples was 0.602 and for the intervention samples was 0.598. In the top 1000 probes, detailed in the heat map, the average beta value for the control samples was 0.788 and for the intervention samples was 0.764. A total of 927 of the top 1000 differentially methylated probes showed decreased average methylation in the intervention group relative to control samples, with an average decrease of 3% in methylation per CpG site. Only 73 of the top 1000 probes had increased methylation in the intervention samples rather than the control samples, with an average increase of 6% in methylation.

Individual cord blood samples are plotted on the x-axis, and individual probes on the y-axis. Completely unmethylated probes (beta value of 0) are represented by yellow and completely methylated probes (beta value of 1) are represented as red. The associated dendrogram indicates the relatedness of samples by methylation, with branches closer together more similar than those further apart. The heatmap showed that almost all intervention samples clustered together (97% in cluster 2), with only one sample from the intervention group grouping in cluster 1. This highlights the subtle, yet widespread differential methylation across groups, and suggests that a subset of individuals are most sensitive to the intervention. 

### 3.4. Pathway Analysis

Functional clusters made up of the top 1000 differentially methylated probes were created using DAVID. Six clusters were created based on KEGG and REACTOME pathways. The top three clusters are summarised in Table 3. The top cluster was related to cardiac functioning. The genes from this cluster are involved in pathways relating to cardiomyopathy (KEGG pathways hsa05414, hsa05410, hsa05412) and cardiac muscle contraction (KEGG pathway hsa04260). The second and third clusters were involved in cancer development and immune function.

### 3.5. Replication

**Figure 3:**
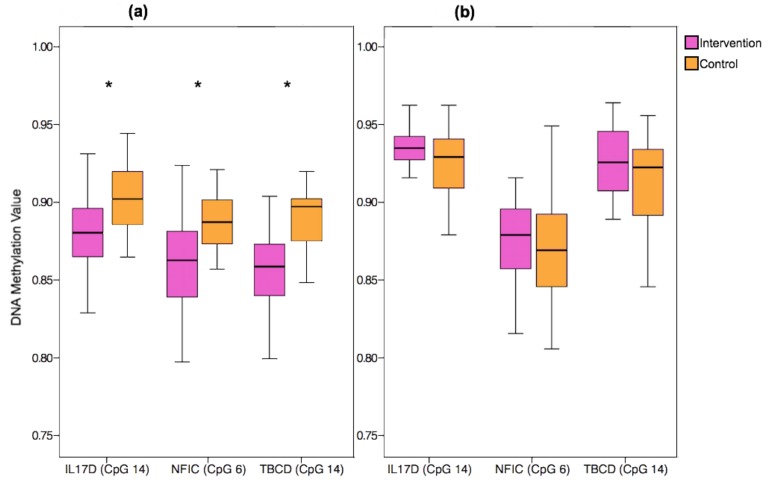
DNA Methylation values for the three selected candidate genes from the ROLO Study split by intervention group (pink) and control group (orange). (**a**) Methylation beta values from the selected candidate genes using the HM850 array (*n* = 60). (**b**) Methylation values from the candidate genes obtained using the Sequenom array (*n* = 60). * = *p* < 0.01. *IL17D:* Interleukin 17D, *NFIC*: Nuclear Factor I C, *TBCD*: Tubulin Folding Cofactor D.

There were no significant differences in the characteristics of the original and replication cohorts (relating to maternal age, weight, BMI, education level, smoking status, gestational weight gain, or glycaemic index in any of the trimesters (*p* > 0.05 for all)). DNA methylation levels at three HM850 probes in *IL17D* (cg18786411, cg05130518, cg00812799), *NFIC* (cg03381209, cg10688516, cg03641241), and *TBCD* (cg16538568, cg21693422, cg00614360) showed differential methylation that reached significance (unadjusted *p* < 0.01). Box plots of selected HM850 probes from the three candidate genes and the DNA methylation values are displayed in Figure 3a. In the replication of the findings for *IL17D*, *NFIC*, and *TBCD* in the additional sample of ROLO offspring, however, neither the direction nor the magnitude of these associations were consistently replicated (see Figure 3b and Supplementary Tables S2 and S3). In this additional group analysed with the Sequenom array, there was some evidence of differential methylation of CpGs located in *TBCD*, however, they were in the opposite direction to the original sample set, being significantly higher in the intervention group relative to the control group. Some sex-differences were seen in the methylation values (see Supplementary Tables S4 and S5), however, these findings were not consistent between the original cohort and the replication cohort. 

### 3.6. Cell-Type Analysis

We investigated the differences in cell composition in the samples between intervention and control groups and also the association of the cell types with the methylation analysis. There were some differences between proportions of cell types in the control and intervention groups (Table 4). The relative proportion of each cell type was measured and higher levels of B lymphocytes, CD4T cells, natural killer cells, and nucleated red blood cells were found in the intervention group. When we looked at principal component analysis, the cell types were significantly associated with PC 1 and PC 2, alongside membership of the intervention group (see Supplementary Table S6). Hierarchical clustering of the top 1000 unadjusted probes was carried out controlling for the cell types (Supplementary Figure S1). This resulted in three methylation clusters associated with the intervention (*p* ≤ 0.01, Fisher’s Exact Test). Cluster 2 contained only participants in the control group, while cluster 1 and 3 had significantly higher proportions of participants from the intervention groups (*p* ≤ 0.01, Fisher’s Exact Test). Again, neither infant sex nor macrosomia tracked with these clusters, however, maternal BMI categories were significantly different between the three clusters (*p* = 0.03, Fisher’s Exact Test), with a significantly higher proportion of mothers in the normal BMI category in cluster 2, compared to cluster 1 or 3 (Figure S1). 

## 4. Discussions

We identified preliminary evidence of widespread, yet subtle changes in the neonatal methylome as a result of a dietary intervention during pregnancy. Our results indicate subtle but pervasive differential methylation across the intervention and control groups, with our cluster analysis suggesting that a subset of individuals are most sensitive to the intervention. We found no associations between birth weight or maternal weight/BMI and the neonatal methylome, however, maternal BMI categories appeared to track alongside some patterns of DNA methylation that were highlighted in the two clusters. Previous research on the ROLO study identified different phenotypes that were more responsive to the intervention [26]. These findings suggest that as maternal BMI, particularly in the obese category, can be indicative of metabolic disorder [27], this could be a vital factor in an individual’s response to interventions aimed at altering the maternal metabolic environment. Our findings suggest that the obese metabolic maternal environment, coupled with the altered environment due to the intervention, may potentially result in unique DNA methylation patterns in the offspring. 

Despite these provocative findings, with this sample size, the analysis was not powered to detect changes in maternal and fetal outcomes as a result of the intervention, and to relate these to methylation. However, in the complete cohort (*n* = 759), the mothers in the intervention group had less gestational weight-gain and less glucose intolerance [14]. In terms of diet, the intervention group significantly reduced their glycaemic index while also reducing energy intake, taking in a higher percentage of energy from protein and also increasing their fibre intake [17]. These changes may contribute to the modest observed variation in the neonatal methylome. In terms of adherence to the intervention, a subgroup of participants (*n* = 212) completed a compliance questionnaire and 80.2% reported following the diet “most/all of the time”. Analysis of the ROLO cohort found phenotypic changes of reduced thigh circumference in neonates born to mothers in the intervention group [28]. Other studies investigating a low glycaemic index dietary intervention found similar results, with the LIMIT trial reporting reduced incidence of macrosomia but no other neonatal anthropometric measures [29,30] and the UPBEAT trial reporting no impact of the low GI diet on neonatal measures but reduced adiposity in the offspring at 6 months of age [31,32]. These alterations in offspring body composition as a result of a low GI diet may potentially be mediated by epigenetic mechanisms. 

We identified potential pathways that were associated with genes impacted by the low glycaemic index dietary intervention. The genes that were implicated were involved in cardiac and immune functioning. Spikes in blood glucose levels, or high postprandial glycaemia, trigger metabolic responses and a low glycaemic index diet aims to decrease this occurrence and provide a more controlled maternal environment for the development of the fetus. While there is a paucity of research related to these pathways and the transferable effects of maternal low glycaemic index diets in pregnancy, a meta-analysis in non-pregnant cohorts reported that low glycaemic index diets were independently associated with reduced risk of certain diseases, such as heart disease and certain cancers [33]. Research in a European cohort has shown that high dietary glycaemic index in pregnancy was linked with increased markers of the metabolic syndrome in young adult offspring [34] and, additionally, a pilot randomised controlled trial carried out in Australia observed that a low glycaemic index diet during pregnancy influenced offspring arterial wall thickness in early childhood [35]. Our findings may aid in elucidating the mechanisms behind this. 

A study in 2010 found that placental DNA methylation was associated with the mother’s glycaemia during pregnancy [36]. The study reported adaptations in DNA methylation of the leptin gene in mothers with gestational impaired glucose tolerance, which suggested that the epigenetic profile of leptin may be influenced by maternal plasma glucose levels. We observed differential methylation levels of CpG sites located on the following genes: *IL17D* (a cytokine), *NFIC* (involved in gene expression, transcription, and DNA binding), and *TBCD* (a tubulin-folding protein involved in binding). These candidate genes have yet to be implicated in mechanisms related to a dietary intervention and glycaemic index diet. However, it is important to note that replication of these findings in an additional sample set from our cohort was unsuccessful. This may be due to the small sample size in this analysis, minor phenotypic differences in the selected sample set not accounted for in analysis, or this may potentially be due to the use of two different DNA methylation analysis techniques (Sequenom and HM850). There are many challenges related to the diversity of DNA methylation profiling techniques available, all of which have their own strengths and limitations [37]. Comparing and replicating results obtained using different techniques can prove difficult and this is a challenge that will need to be overcome for future DNA methylation and EWAS studies. Nevertheless, our attempts at replication were important to put the discovery sample findings into perspective and this needs to be considered in similar-sized analyses for other exposures and outcomes. Upon the addition of the cell type analysis, we found differences in the cell composition between the samples from infants born into the control and intervention groups. This may aid in explaining the findings of the subtle differences in methylation status between the two groups. If the intervention is impacting cell type composition, this could have implications for the inflammatory milieu associated with adverse metabolic programming. This has not been reported previously in other studies and represents further avenues of exploration. 

This study had many strengths, including the use of the most powerful and widespread methylation array available, the Illumina Infinum MethylationEPIC BeadChip Array, which allowed the comprehensive investigation of 771,484 CpG sites in the cord blood DNA. Furthermore, we had extensive, detailed information on the participants, both the mother and child, with phenotypic data along with comprehensive food diaries for analysis of the dietary glycaemic index and health and lifestyle information. However, this study was not without limitations. As part of the methodology this analysis excluded cross-reactive probes based on the Human Methylation 450 K array [18], however, in the time since this analysis was carried out a new list of cross-reactive probes has been published for the HM850 array [38], which may impact on the findings. Although sufficient for exploratory analysis, the small sample size may have made it difficult to detect some of the more subtle biological impacts of the intervention and caution should be taken with interpreting the results to apply to a larger population. Certain characteristics, such as the BMI categories, may have tracked with the intervention or control groups, as the analysis was not carried out on the full sample set from the randomised controlled trial. The DNA methylation profiles were derived from cord serum samples collected at birth, which could pose an issue with cell composition and might be the driver of some of the effect. Research has also shown that pathway analyses can be inherently biased due to the array design, which should also be considered in this dataset [39]. Other epigenetic mechanisms, such as microRNA expression or histone modification, which may be more responsive to environmental modulation, could potentially be influenced by this altered maternal environment induced by the low glycaemic index diet, and this represents further avenues of investigation.

These data suggest that exposure to a dietary intervention may impact the epigenome in a widespread but subtle manner, potentially driven by a complex interplay of maternal and offspring effects on the regulation of DNA methylation during fetal development, and this may be important for health and disease. With the hypothesis that higher postprandial glycaemia may be a universal mechanism for disease progression [33], our findings indicate that altering the maternal environment with a low glycaemic index diet could confer a healthy methylome in the neonate. Further research is required with larger cohorts to test this, with independent replication, if possible. Caution should be used in study design and data interpretation with modest sample sizes, in the absence of additional replication in independent samples. 

## 5. Conclusions

This low glycaemic index dietary intervention during pregnancy suggests subtle, yet widespread differential DNA methylation at regions across the offspring’s genome. These data imply that exposure to a dietary intervention may impact the neonatal epigenome during fetal development. There were no independent associations with maternal early pregnancy BMI or birth weight, however, a potential interplay between maternal BMI and the intervention was identified. Genes involved in pathways related to pancreatic and immune function were influenced by the intervention and this may contribute to a further understanding of the epigenetic regulatory mechanisms in utero and how maternal diet may impact this. 

Our findings highlight the importance of intervention studies, while also underlining the need for caution when selecting suitable cohorts and analysis methods and also in interpreting results. Larger studies are required to fully explore interventions in pregnancy to break the cycle of transmission of poor metabolic health from mother to offspring via epigenetic variation.

## Figures and Tables

**Table 1:** Characteristics of the mothers and neonates in the ROLO Study cohort (*n* = 60).

	Intervention		Control		*p*
***n***	30		30		
*Maternal Characteristics*	Mean	SD	Mean	SD	
Mother Age (years)	32.78	4.48	33.91	4.16	0.31
Mother Weight (14 weeks, kg)	75.89	12.03	70.93	11.06	0.10
Maternal BMI (14 weeks, kg/m^2^)	27.72	4.26	25.66	7.74	0.05
3rd Level Education (*n* (%))	15 (50)		20 (66.7)		0.37
Smoking During Pregnancy (*n* (%))	0 (0)		0 (0)		-
Gestational Weight Gain (kg)	11.82	3.50	13.93	5.01	0.09
Daily GI Trimester 1	57.74	3.38	57.51	3.47	0.79
Energy intake Trimester 1 (kcals)	1803.19	308.64	1970.90	468.81	0.11
Daily GI Trimester 2	56.42	3.60	57.35	3.28	0.30
Energy intake Trimester 2 (kcals)	1795.54	440.82	1960.72	383.73	0.13
Daily GI Trimester 3	55.34	3.80	57.37	2.99	0.03 *
Energy intake Trimester 2 (kcals)	1826.38	400.64	2023.79	409.63	0.06
*Maternal BMI Category at Booking Visit* (*n* (%))					
Normal (18.5–24.9 kg/m^2^)	8 (26.7)		17 (56.7)		0.04 *
Overweight (25–29.9 kg/m^2^)	16 (53.3)		10 (33.3)		0.19
Obese (≥30 kg/m^2^)	6 (20.0)		3 (10)		0.47
*Neonatal Characteristics*					
Birth Weight (kg)	4.20	0.62	3.98	0.43	0.12
Macrosomic Neonate (*n* (%))	16 (53.3)		14 (46.7)		0.80
Gestational Age (weeks)	40.33	1.07	40.10	1.17	0.55

Values are Means or Standard Deviation (SD) or as indicated. ROLO: Randomised cOntrol trial of LOw glycaemic index diet versus no dietary intervention to prevent recurrence of fetal macrosomia, BMI: Body Mass Index, GI: Glycaemic Index, Statistical comparisons by student *t*-test and Chi-square tests. * *p* < 0.05.

**Table 2:** Principal Component Analysis of factors during pregnancy influencing DNA methylation levels in offspring.

	Individual	Chip	Chip Position	Infant Sex	RCT Group	Maternal BMI	Maternal Weight (kg)	Maternal Age (years)	Birth Weight (kg)	Gestational Age (weeks)
PC 1 correlation	0.062	0.005	0.405	−0.015	−0.049	0.130	0.097	0.031	0.146	−0.097
PC 1 *p* value	0.638	0.970	0.001 *	0.912	0.713	0.322	0.461	0.813	0.264	0.463
PC 2 correlation	0.198	−0.029	−0.080	−0.228	−0.407	0.098	0.090	0.076	0.113	0.101
PC 2 *p* value	0.130	0.824	0.545	0.080	0.001 *	0.456	0.494	0.565	0.391	0.444
PC 3 correlation	0.153	0.435	−0.487	−0.150	0.048	−0.294	−0.282	0.198	0.137	−0.057
PC 3 *p* value	0.243	0.001 *	0.000 *	0.251	0.718	0.022	0.029	0.129	0.297	0.663
PC 4 correlation	0.457	−0.078	0.131	0.185	0.088	0.024	0.009	0.035	−0.102	0.029
PC 4 *p* value	0.000 *	0.555	0.320	0.156	0.502	0.858	0.946	0.792	0.437	0.824
PC 5 correlation	−0.051	−0.294	−0.316	0.059	0.150	−0.204	−0.110	0.053	−0.259	0.095
PC 5 *p* value	0.696	0.023	0.014	0.656	0.253	0.118	0.404	0.687	0.046	0.472
PC 6 correlation	−0.085	0.179	−0.047	0.054	−0.152	−0.027	0.051	0.023	−0.212	−0.142
PC 6 *p* value	0.518	0.172	0.724	0.684	0.247	0.840	0.700	0.864	0.105	0.281
PC 7 correlation	−0.358	0.312	0.115	−0.289	0.237	0.023	0.026	−0.044	−0.007	0.016
PC 7 *p* value	0.005 *	0.015	0.380	0.025	0.068	0.862	0.841	0.741	0.958	0.901
PC 8 correlation	0.528	−0.274	0.187	0.112	−0.096	0.103	0.037	0.238	−0.290	−0.006
PC 8 *p* value	0.000 *	0.034	0.153	0.394	0.464	0.435	0.778	0.067	0.025	0.966
PC 9 correlation	−0.070	−0.036	−0.038	0.071	0.046	−0.187	−0.257	0.258	−0.115	0.251
PC 9 *p* value	0.596	0.784	0.775	0.592	0.729	0.153	0.048	0.046	0.381	0.053
PC 10 correlation	0.103	−0.208	−0.132	−0.564	−0.028	−0.012	0.062	−0.079	0.289	−0.071
PC 10 *p* value	0.434	0.111	0.314	0.000 *	0.829	0.928	0.638	0.548	0.025	0.589

Table displaying correlation coefficients, directions of correlation, and *p*-values between principal components and various clinical parameters. Shaded boxes indicate direction of significant correlations between principal components and clinical parameters (orange: positive correlations, blue: negative correlation). Correlation coefficients were calculated using Pearson correlations. PC: Principal Component, RCT: Randomised Controlled Trial * Significant at *p* < 0.05.

**Table 3:** Gene functional clusters of the 1000 highest ranked probes associated with the ROLO intervention/control group.

	Pathway	Function	Count	*p*	Benjamini
Cluster 1: Cardiac Functioning					
ES: 0.78	KEGG	Dilated cardiomyopathy	7	0.11	0.72
	KEGG	Cardiac muscle contraction	6	0.14	0.7
	KEGG	Hypertrophic cardiomyopathy (HCM)	6	0.18	0.74
	KEGG	Arrhythmogenic right ventricular cardiomyopathy (ARVC)	5	0.28	0.79
Cluster 2: Cancer Formation					
ES: 0.72	KEGG	ErbB signalling pathway	7	0.087	0.75
	KEGG	Non-small cell lung cancer	5	0.12	0.7
	KEGG	Glioma	3	0.66	0.94
Cluster 3: Immune Functioning					
ES: 0.69	KEGG	T cell receptor signalling pathway	8	0.087	0.71
	KEGG	Natural killer cell mediated cytotoxicity	8	0.19	0.73
	KEGG	Fc epsilon R1 signalling pathway	4	0.53	0.92

Clusters created using DAVID (Database for Annotation, Visualization and Integrated Discovery-http://david.abcc.ncifcrf.gov/) Gene Functional Classification Tool using Kyoto Encyclopedia of Genes and Genomes (KEGG) and REACTOME pathways. ES: Enrichment Score, Benjamini: Benjamini-Hochberg False-discovery-rate method [22] globally corrected enrichment *p*-values that control family-wide false discovery ≤ 0.05.

**Table 4:** Relative proportion of cell types in ROLO cord blood samples (*n* = 60).

Cell Type	Total Group		Intervention		Control		*p*
	Median	IQR	Median	IQR	Median	IQR	
B cells	0.025	0.040	0.040	0.063	0.020	0.030	0.014 *
CD4T	0.050	0.038	0.050	0.053	0.030	0.033	0.02 *
CD8T	0.000	0.008	0.000	0.010	0.000	0.000	0.048 *
Granulocytes	0.825	0.185	0.800	0.258	0.855	0.140	0.042 *
Monocytes	0.015	0.048	0.030	0.063	0.010	0.020	0.123
NK cells	0.030	0.030	0.040	0.043	0.030	0.030	0.006 *
nRBC	0.040	0.048	0.055	0.060	0.030	0.043	0.026 *

B cells: B lymphocyte cells, CD4T: CD4+ helper T cells, CD8T: Cytotoxic T lymphocyte cells, NK: Natural Killer cytotoxic lymphocyte cells, nRBC: Nucleated red blood cells, IQR: Interquartile range. * Significant at *p* < 0.05, *p*-values calculated using Mann-Whitney U tests.

## Supplementary Materials

The following are available online at http://www.mdpi.com/2072-6643/10/4/455/s1, Table S1: Sequenom Assays and primer design for validation, Table S2: HM850 probes—Beta values and differences, Table S3: Sequenom Data: Total Group (*n* = 60) methylation values and differences, Table S4: Sequenom Data: Males only (*n* = 30) methylation values and differences, Table S5: Sequenom Data: Female only (*n* = 30) descriptives and methylation values, Table S6: Principal Component Analysis of cell-type influencing DNA methylation levels in offspring, Supplementary Figure S1: Hierarchical clustering and heatmap of HM850 methylation values of the top 1000 probes associated with intervention/control.
