# Supplementary material for: A Low Glycaemic Index Diet in Pregnancy Induces DNA Methylation Variation in Blood of Newborns: Results from the ROLO Randomised Controlled Trial
Source: Nutrients. 2018 Apr 6;10(4):455. doi: 10.3390/nu10040455 (PMC5946240; doi:10.3390/nu10040455)
Supplement: Supplementary file 1 [file nutrients-10-00455-s001.pdf]

Supplementary tables:

Table S1. Sequenom Assays and primer design for validation.

Candidate Gene	Forward Primer (5′–3′)	Reverse Primer (5′–3′)	Amplicon Size (bp)	HM850 Probe name	Genomic CpG Location*	Assay CpG Identity
IL17D	TTTTAGGTAGGTATTTGGGGTA TTT	CCTCATTCTATTCTAATAACACCTATA AAC	354	cg18786411	CpG13:21,295,528 hg19	IL17D CpG14
NFIC	GGGTTTTATTATGTTGGTTAGGT TG	AAATCCCCACACAAATACATTCATA	266	cg03641241	CpG19:3,392,471 hg19	NFIC CpG6
TBCD	TTGGAAATTTAGATGTTAGTGG AAT	CCCACATAACCCAAAAAAAATAACT	392	cg16538568	CpG17:80,823,310 hg19	TBCD CpG14

* named as CpG"chromosome number location":"genomic numerical location" "UCSC human genome build".

Table S2. HM850 probes—Beta values and differences.

Corresponding-HM850 Probes	Intervention (Beta Value)	Control (Beta Value)	Mean % Difference	Mean Difference	*P*-value
cg18786411 HM850 (IL17D CpG14)	0.88 (0.02)	0.90 (0.02)	-2.27	-0.02	<0.00001
cg03641241 HM850 (NFIC CpG6)	0.86 (0.04)	0.89 (0.02)	-3.02	-0.03	<0.00001
cg16538568 HM850 (TBCD CpG14)	0.85 (0.04)	0.89 (0.02)	-3.70	-0.04	<0.00001

Table S3. Sequenom Data: Total Group (n = 60) methylation values and differences.

Gene & CpG	Intervention	Control	Mean Difference	Mean % Difference	*P*-value
**IL17D CpG 1&13**	0.56 (0.03)	0.56 (0.04)	0.002	0.17	0.842
**IL17D CpG 2**	0.15 (0.04)	0.13 (0.5)	0.014	1.36	0.228
**IL17D CpG 8&9**	0.55 (0.04)	0.55 (0.06)	0.008	0.82	0.524
**IL17D CpG 10&11**	0.64 (0.04)	0.64 (0.07)	0.008	0.84	0.558
**IL17D CpG 12**	0.56 (0.12)	0.55 (0.10)	0.016	1.61	0.615
**IL17D CpG 14**	0.94 (0.01)	0.93 (0.03)	0.011	1.12	0.090
**IL17D CpG 15**	0.89 (0.03)	0.88 (0.04)	0.006	0.59	0.506
**IL17D CpG 16**	0.68 (0.13)	0.70 (0.13)	-0.016	-1.59	0.963[a]
**IL17D (mean)**	0.62 (0.03)	0.61 (0.03)	0.008	0.82	0.262

**NFIC CpG 1**	0.90 (0.03)	0.93 (0.03)	-0.005	-0.49	0.337[a]
**NFIC CpG 2**	0.88 (0.06)	0.89 (0.05)	-0.003	-0.28	0.825[a]
**NFIC CpG 3**	0.87 (0.02)	0.88 (0.02)	-0.004	-0.37	0.533
**NFIC CpG 4**	0.87 (0.03)	0.86 (0.04)	0.007	0.68	0.648[a]
**NFIC CpG 5**	0.98 (0.02)	0.99 (0.01)	-0.003	-0.35	0.829[a]
**NFIC CpG 6**	0.87 (0.05)	0.86 (0.05)	0.003	0.30	0.818
**NFIC (mean)**	0.90 (0.03)	0.90 (0.03)	-0.001	-0.08	0.912
**TBCD CpG 2&4&11&12**	0.89 (0.08)	0.83 (0.08)	0.051	5.12	0.028
**TBCD CpG 5&6&7**	0.93 (0.03)	0.92 (0.05)	0.008	0.75	0.486
**TBCD CpG 13**	0.82 (0.09)	0.78 (0.12)	0.044	4.39	0.194
**TBCD CpG 14**	0.92 (0.03)	0.89 (0.08)	0.032	3.18	0.188[a]
**TBCD (mean)**	0.89 (0.05)	0.85 (0.06)	0.040	4.03	0.020

P-values calculated using independent samples T-test unless otherwise stated. [a] Mann-Whitney U test used to calculate *p*-value.

Table S4. Sequenom Data: Males only (n = 30) methylation values and differences.

**Gene & CpG**	**Intervention**	**Control**	**Mean Difference**	**Mean % Difference**	***P*-value**
**IL17D CpG 1&13**	0.57 (0.03)	0.56 (0.04)	0.004	0.43	0.745
**IL17D CpG 2**	0.15 (0.03)	0.13 (0.05)	0.016	1.62	0.275
**IL17D CpG 8&9**	0.55 (0.03)	0.53 (0.06)	0.019	1.87	0.268
**IL17D CpG 10&11**	0.62 (0.02)	0.63 (0.08)	-0.004	-0.37	0.866
**IL17D CpG 12**	0.55 (0.13)	0.54 (0.12)	0.008	0.75	0.883
**IL17D CpG 14**	0.93 (0.01)	0.92 (0.04)	0.012	1.21	0.231
**IL17D CpG 15**	0.88 (0.03)	0.87 (0.04)	0.009	0.92	0.445
**IL17D CpG 16**	0.68 (0.13)	0.70 (0.14)	-0.017	-1.73	0.821[a]
**IL17D (mean)**	0.62 (0.02)	0.61 (0.03)	0.013	1.32	0.144
**NFIC CpG 1**	0.90 (0.01)	0.90 (0.03)	0.002	0.18	0.813[a]
**NFIC CpG 2**	0.90 (0.03)	0.89 (0.05)	0.018	1.75	0.334[a]
**NFIC CpG 3**	0.88 (0.02)	0.88 (0.02)	-0.003	-0.27	0.732
**NFIC CpG 4**	0.88 (0.03)	0.85 (0.05)	0.026	2.57	0.102[a]
**NFIC CpG 5**	0.99 (0.01)	0.99 (0.01)	0.005	0.51	0.298[a]

**NFIC CpG 6**	0.88 (0.02)	0.85 (0.05)	0.025	2.55	0.099
**NFIC (mean)**	0.91 (0.02)	0.89 (0.03)	0.012	1.22	0.163
**TBCD CpG 2&4&11&12**	0.89 (0.07)	0.81 (0.09)	0.084	8.42	0.010
**TBCD CpG 5&6&7**	0.92 (0.02)	0.92 (0.06)	0.003	0.31	0.857
**TBCD CpG 13**	0.84 (0.08)	0.77 (0.11)	0.076	7.61	0.106
**TBCD CpG 14**	0.91 (0.03)	0.89 (0.07)	0.029	2.90	0.316[a]
**TBCD (mean)**	0.91 (0.05)	0.84 (0.07)	0.067	6.66	0.007

P-values calculated using independent samples T-test unless otherwise stated. [a] Mann-Whitney U test used to calculate *p*-value.

Table S5: Sequenom Data: Female only (n=30) descriptives and methylation values

Gene & CpG	Intervention	Control	Mean Difference	Mean % Difference	*P*-value
**IL17D CpG 1&13**	0.56 (0.03)	0.56 (0.02)	-0.001	-0.13	0.909
**IL17D CpG 2**	0.15 (0.05)	0.14 (0.04)	0.009	0.91	0.617
**IL17D CpG 8&9**	0.56 (0.05)	0.57 (0.04)	-0.010	-1.01	0.567
**IL17D CpG 10&11**	066 (0.04)	0.65 (0.03)	0.015	1.52	0.316
**IL17D CpG 12**	0.57 (0.10)	0.55 (0.06)	0.022	2.16	0.585
**IL17D CpG 14**	0.94 (0.01)	0.93 (0.01)	0.007	0.66	0.195
**IL17D CpG 15**	0.89 (0.03)	0.89 (0.03)	-0.002	-0.16	0.904
**IL17D CpG 16**	0.68 (0.14)	0.70 (0.12)	-0.015	-1.55	0.820 [a]
**IL17D (mean)**	0.63 (0.03)	0.63 (0.03)	0.000	0.01	0.991
**NFIC CpG 1**	0.90 (0.03)	0.91 (0.04)	-0.014	-1.37	0.274 [a]
**NFIC CpG 2**	0.87 (0.08)	0.89 (0.05)	-0.022	-2.25	0.595 [a]
**NFIC CpG 3**	0.87 (0.03)	0.88 (0.02)	-0.005	-0.50	0.605
**NFIC CpG 4**	0.87 (0.03)	0.88 (0.04)	-0.020	-1.96	0.160 [a]
**NFIC CpG 5**	0.98 (0.03)	0.99 (0.01)	-0.012	-1.24	0.527 [a]
**NFIC CpG 6**	0.85 (0.07)	0.88 (0.04)	-0.025	-2.46	0.289
**NFIC (mean)**	0.89 (0.04)	0.91 (0.03)	-0.016	-1.63	0.235
**TBCD CpG 2&4&11&12**	0.88 (0.09)	0.87 (0.07)	0.007	0.74	0.824
**TBCD CpG 5&6&7**	0.93 (0.03)	0.92 (0.03)	0.010	1.01	0.429
**TBCD CpG 13**	0.80 (0.09)	0.79 (0.14)	0.014	1.44	0.780

**TBCD CpG 14**	0.93 (0.10)	0.90 (0.10)	0.036	3.57	0.346 [a]
**TBCD (mean)**	0.88 (0.06)	0.87 (0.04)	0.004	0.43	0.854

P-values calculated using independent samples T-test unless otherwise stated. [a]Mann-Whitney U test used to calculate *p*-value

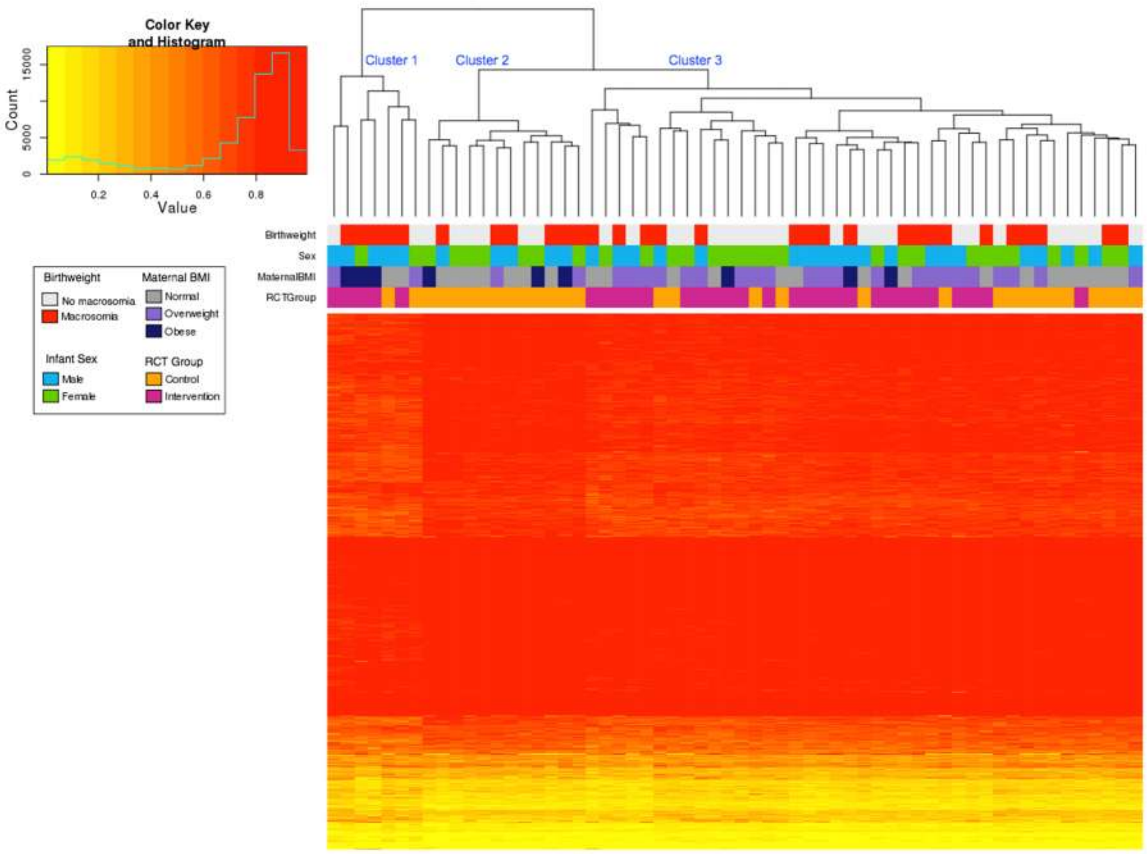

Figure S1. Hierarchical clustering and heatmap of HM850 methylation values of the top 1000 probes associated with intervention/control. Controlled for sex, gestational age, chip, chip position, and cell types (B cells, CD4T, CD8T, granulocytes, monocytes, NK cells, and nRBCs). The histogram depicts the distribution of methylation levels across all samples and probes, the beta value is plotted on the x-axis and number of probes on the y-axis. Individual cord blood samples are plotted on the x-axis, and individual probes on the y-axis. Completely unmethylated probes (beta value of 0) are represented by yellow and completely methylated probes (beta value of 1) are represented as red. The associated dendrogram indicates the relatedness of samples by methylation, with branches closer together more similar than those further apart.
